# Supplementary material for: Asymmetric Dinuclear Lanthanide(III) Complexes from the Use of a Ligand Derived from 2-Acetylpyridine and Picolinoylhydrazide: Synthetic, Structural and Magnetic Studies
Source: Molecules. 2020 Jul 10;25(14):3153. doi: 10.3390/molecules25143153 (PMC7397153; doi:10.3390/molecules25143153)
Supplement: Supplementary file 1 [file molecules-25-03153-s001.pdf]

molecules

MDPI

Supplementary Materials

Asymmetric Dinuclear Lanthanide(III) Complexes from the Use of a Ligand Derived from 2-Acetylpyridine and Picolinoylhydrazide: Synthetic, Structural and Magnetic Studies†

Diamantoula Maniaki [1], Panagiota S. Perlepe [2,3], Evangelos Pilichos [1], Sotirios Christodoulou [4], Mathieu Rouzières [2], Pierre Dechambenoit [2,*], Rodolphe Clérac [2,*] and Spyros P. Perlepes [1,5,*]

1 Department of Chemistry, University of Patras, 265 04 Patras, Greece; dia.maniaki@gmail.com (D.M.); pilvag@gmail.com (E.P.)

2 Univ. Bordeaux, CNRS, Centre de Recherche Paul Pascal, UMR 5031, 336 00, Pessac, France; panagiota.perlepe@crpp.cnrs.fr (P.S.P.); mathieu.rouzieres@crpp.cnrs.fr (M.R.)

3 Univ. Bordeaux, CNRS, ICMCB, UMR 5026, 336 00, Pessac, France

4 ICFO-Institut de Ciencies Fotoniques, The Barcelona Institute of Nanoscience and Nanotechnology, Castelldefels, 088 60 Barcelona, Spain; Sotirios.Christodoulou@alumni.icfo.eu (S.C.)

5 Foundation for Research and Technology-Hellas (FORTH), Institute of Chemical Engineering Sciences (ICE-HT), Platani, P.O. Box 1414, 265 04 Patras, Greece

* Correspondence: pierre.dechambenoit@crpp.cnrs.fr (P.D.); rodolphe.clerac@crpp.cnrs.fr (R.C.); perlepes@patreas.upatras.gr (S.P.P.); Tel: +33-556845671 (P.D.); +33-556845650 (R.C.); +30-2610-996730 (S.P.P.)

† This article is dedicated to the memory of Professor Kyriakos Riganakos, an excellent academician, a great food chemistry scientist and a precious friend.

Received: 25 May 2020; Accepted: 5 July 2020; Published: date

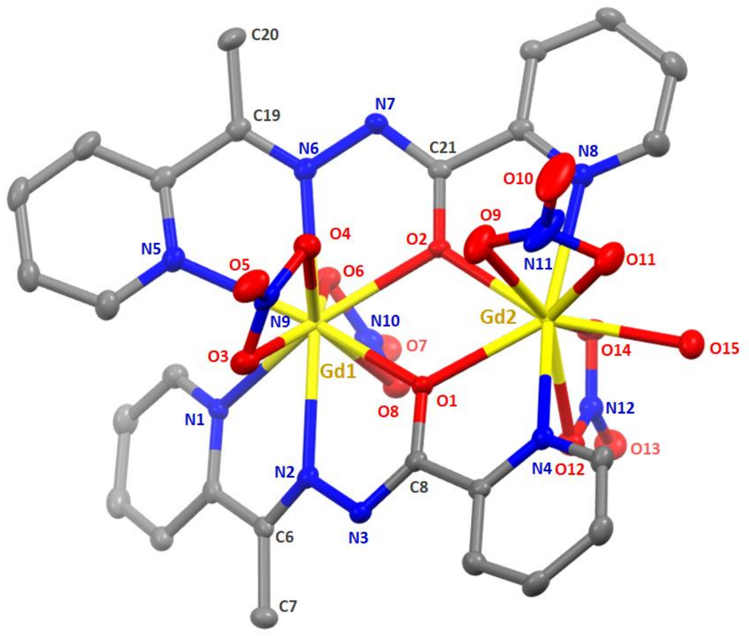

Figure S1. Crystal structure of [$Gd_2(NO_3)_4(L)_2(H_2O)$] as found in **1**·2MeOH·2H_2O at 120 K. Thermal ellipsoids are depicted at 50% probability level. Hydrogen atoms and solvent molecules are omitted for clarity.

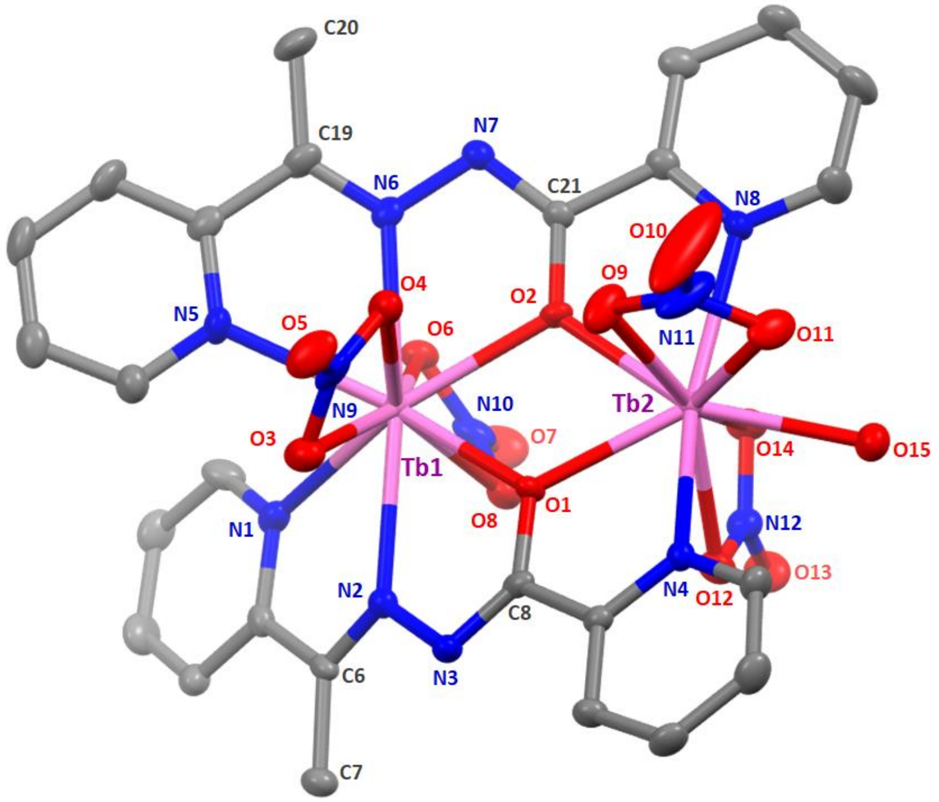

Figure S2. The molecule [$Tb_2(NO_3)_4(L)_2(H_2O)$] that is present in the crystal structure of **2**·2MeOH·1.5H_2O. Thermal ellipsoids are depicted at 50% probability level. Hydrogen atoms are omitted for clarity.

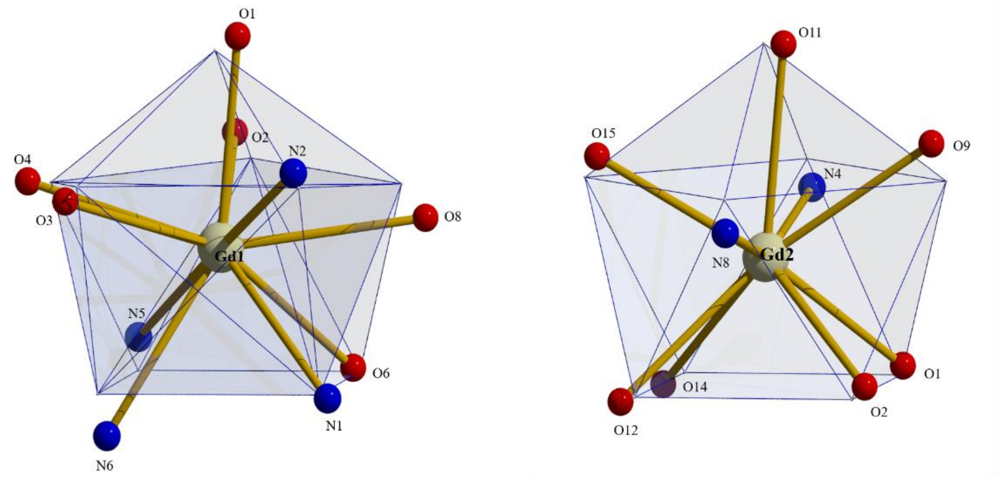

Figure S3. Spenocoronal and spherical capped square antiprismatic coordination geometries of Gd1 and Gd2, respectively, in the structure of **1**·2MeOH·2H_2O. The plotted polyhedra represent the ideal, best-fit polyhedra using the program SHAPE.

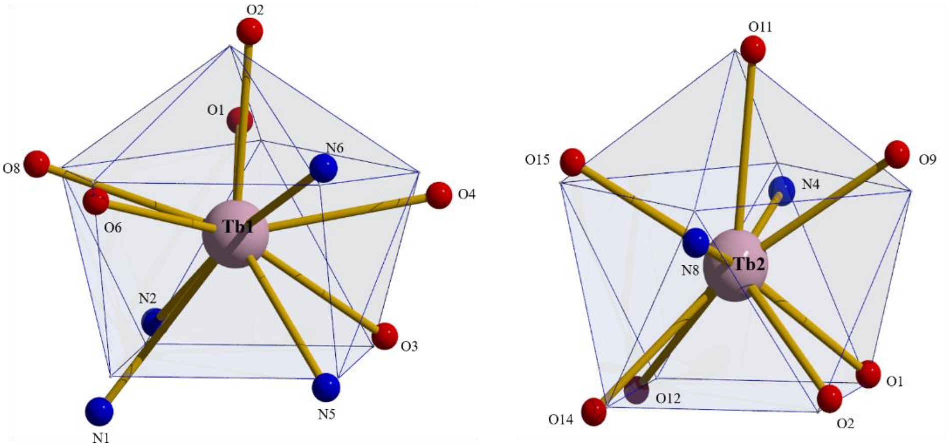

Figure S4. Spenocoronal and spherical capped square antiprismatic coordination geometries of Tb1 and Tb2, respectively, in the structure of **2**·2MeOH·1.5H_2O. The plotted polyhedra represent the ideal, best-fit polyhedra using the program SHAPE.

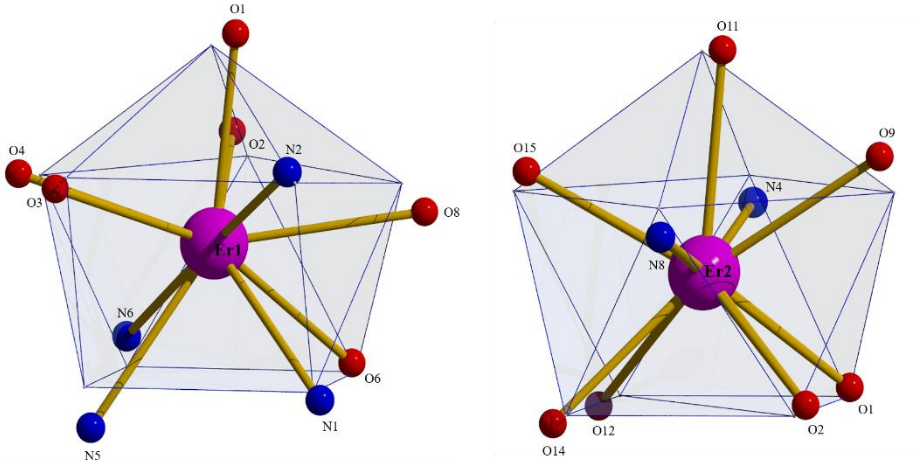

Figure S5. Spenocoronal and spherical capped square antiprismatic coordination geometries of Er1 and Er2, respectively, in the structure of **4**·3MeOH·0.5H_2O. The plotted polyhedra represent the ideal, best-fit polyhedra using the program SHAPE.

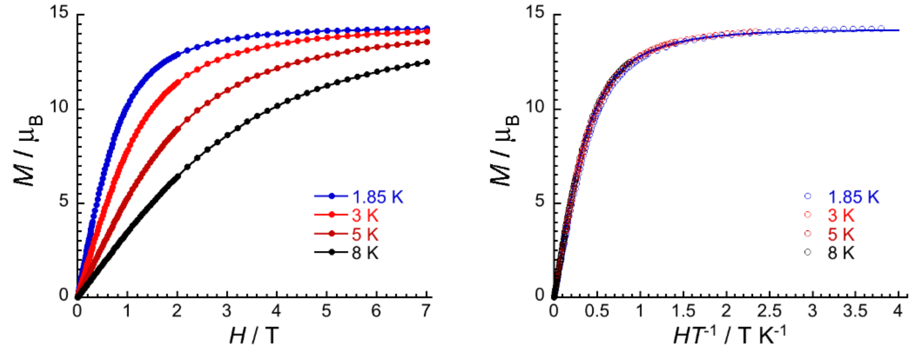

Figure S6. (Left) Field dependence of magnetization for **1**·2MeOH·2H_2O at the temperatures indicated, scanning at 100 – 400 Oe·min^{-1} for $H < 1$ T and 500 – 2500 Oe·min^{-1} for $H > 1$ T. Solid lines are visual guides. (Right) Field dependence of reduced magnetization at the temperatures indicated. Solid line represents the best fit of the *M* vs. *H*/*T* data to the sum of two $S = 7/2$ Brillouin functions.

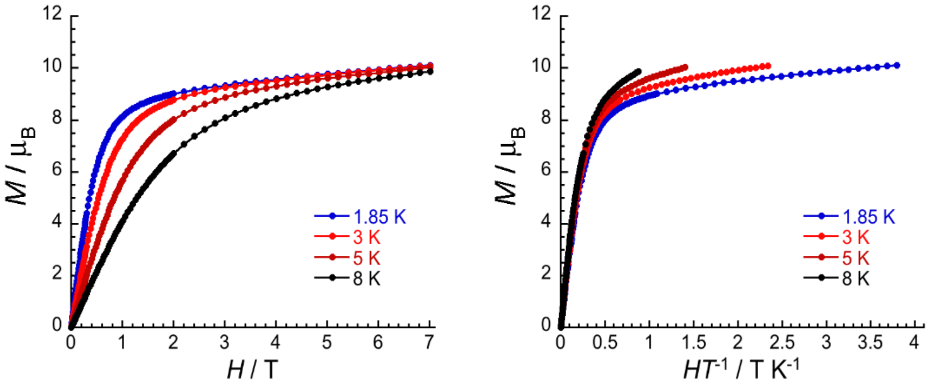

Figure S7. (Left) Field dependence of magnetization for **2**·2MeOH·1.5H_2O at the temperatures indicated, scanning at 100 – 400 Oe·min^{-1} for $H < 1$ T and 500 – 2500 Oe·min^{-1} for $H > 1$ T. (Right) Field dependence of reduced magnetization at the temperatures indicated. Solid lines are guides for the eye.

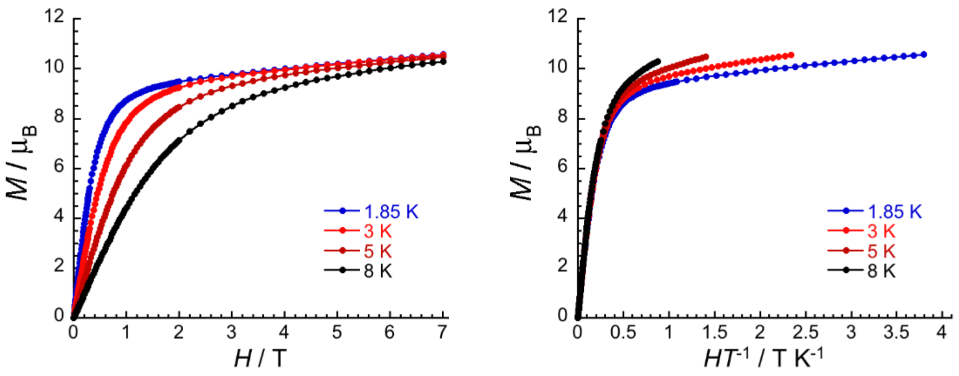

Figure S8. (Left) Field dependence of magnetization for **3**·2.5MeOH at the temperatures indicated, scanning at 100 – 400 Oe·min^{-1} for $H < 1$ T and 500 – 2500 Oe·min^{-1} for $H > 1$ T. (Right) Field dependence of reduced magnetization at the temperatures indicated. Solid lines are guides for the eye.

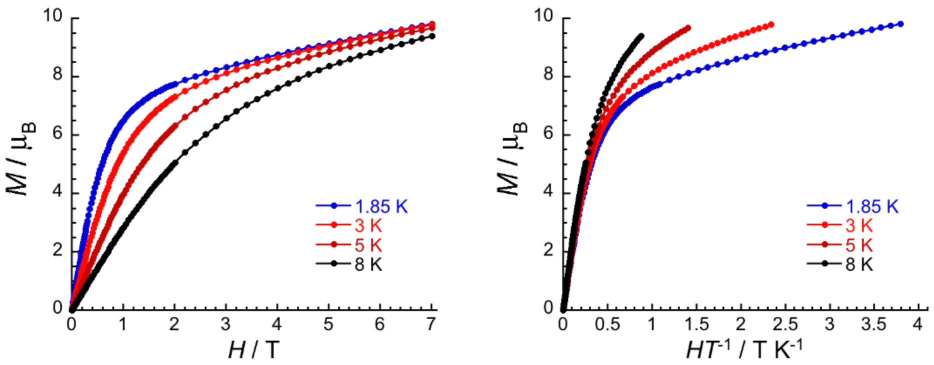

Figure S9. (Left) Field dependence of magnetization for **4**·3MeOH·0.5H$_2$O at the temperatures indicated, scanning at 100–400 Oe·min^{-1} for $H < 1$ T and 500 – 2500 Oe·min^{-1} for $H > 1$ T. (Right) Field dependence of reduced magnetization at the temperatures indicated. Solid lines are guides for the eye.

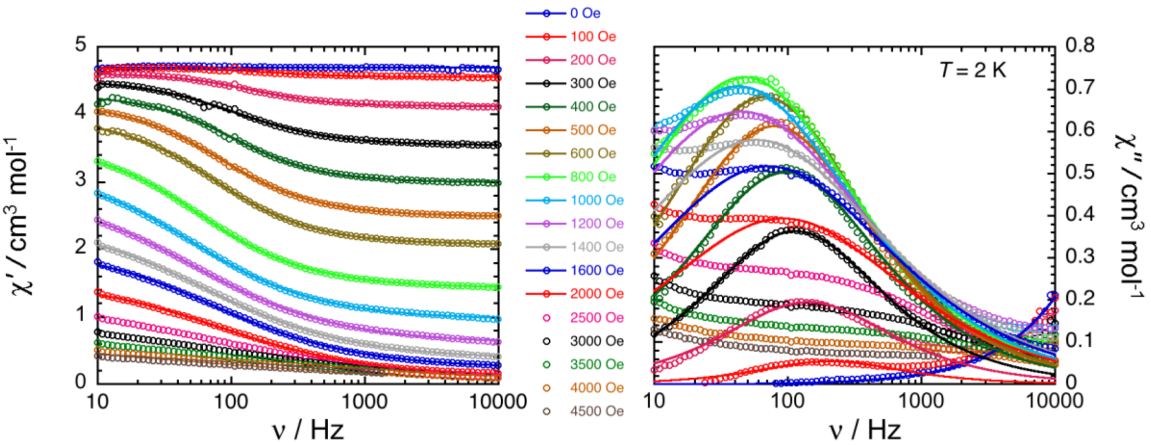

Figure S10. Frequency dependence of the real (χ', left) and imaginary (χ'', right) parts of the ac susceptibility for **3**·2.5MeOH collected at 2 K and varying dc fields. Solid lines are the best fits obtained with the Debye generalized model.

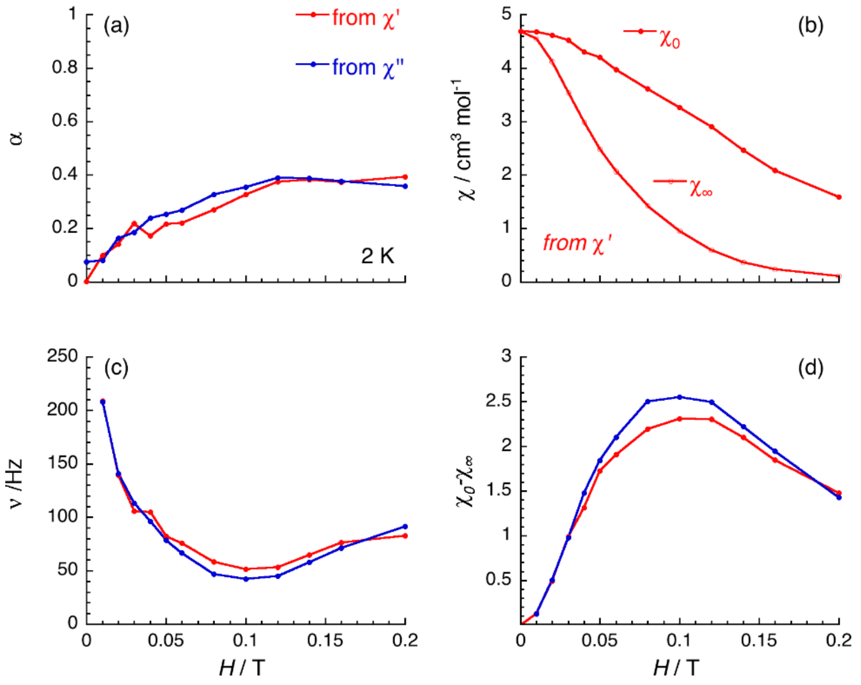

Figure S11. Field dependence of the parameters α (a), χ_0 and χ_∞ (b), ν (c) and χ_0-χ_∞ (d) between 0 and 0.2 T deduced from the generalized Debye fit of the frequency dependence of the real (χ') and imaginary (χ'') components of the ac susceptibility at 2 K, shown in Figure S10, for **3**·2.5MeOH. Solid lines are visual guides.

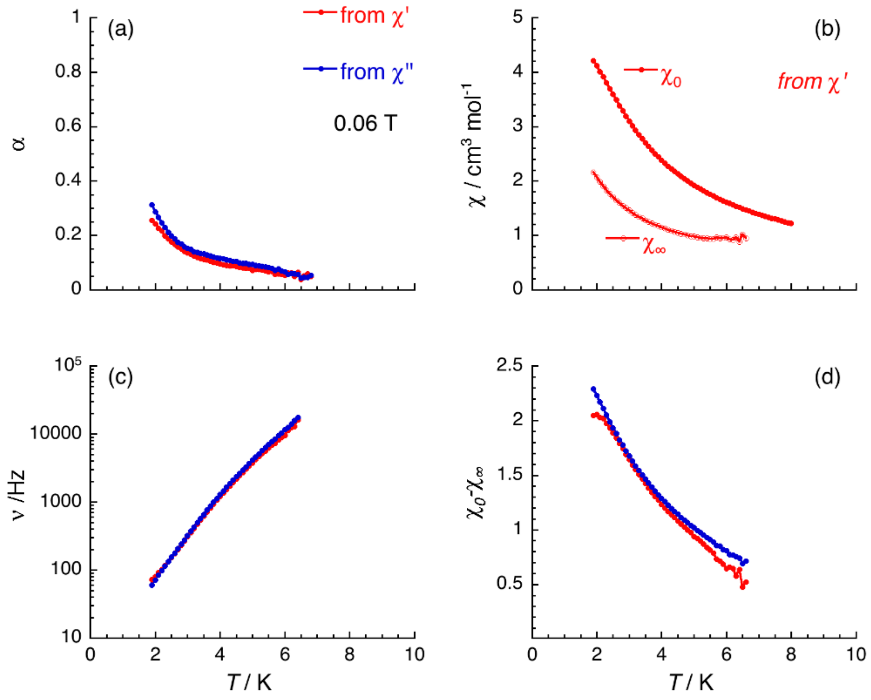

Figure S12. Temperature dependence of the parameters α (a), χ_0 and χ_∞ (b), ν (c) and χ_0-χ_∞ (d) between 1.8 and 8 K deduced from the generalized Debye fit of the frequency dependence of the real (χ') and imaginary (χ'') components of the ac susceptibility at 0.06 T, shown in Figure 8 of the main text, for **3**·2.5MeOH. Solid lines are visual guides.

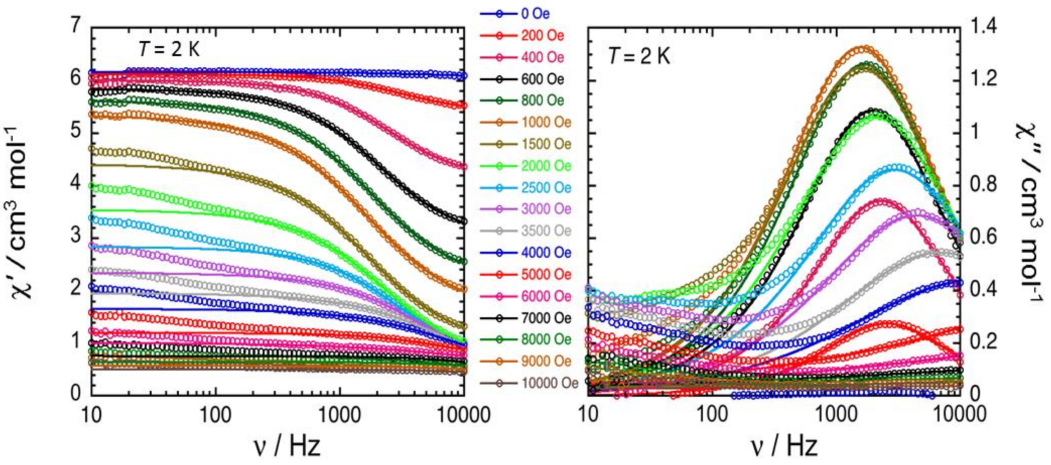

Figure S13. Frequency dependence of the real (χ', left) and imaginary (χ'', right) parts of the ac susceptibility for **4**·3MeOH·0.5H_2O collected at 2 K and varying dc fields. Solid lines are the best fits obtained with the Debye generalized model.

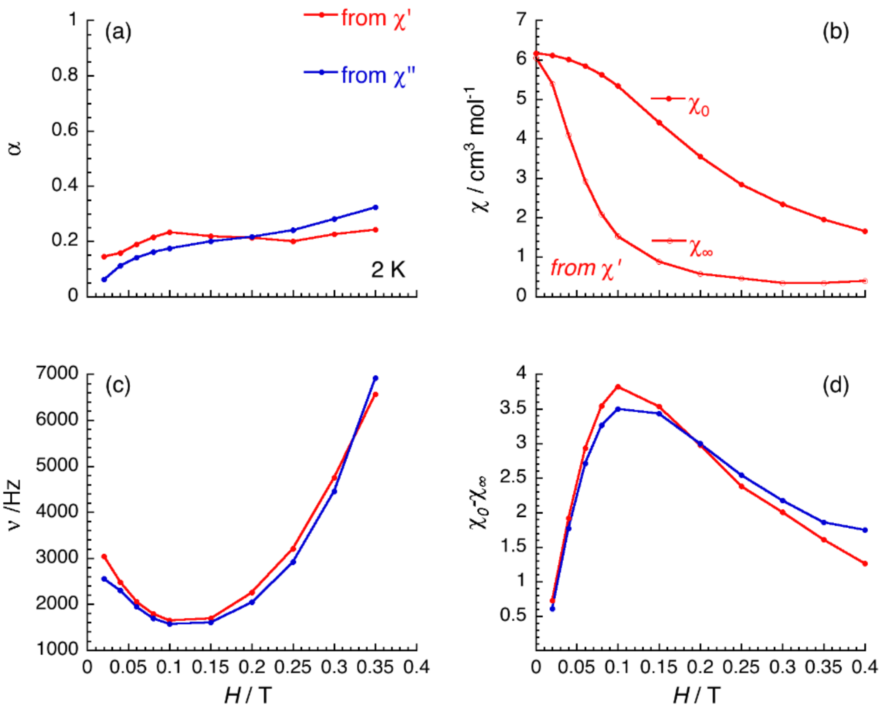

Figure S14. Field dependence of the parameters α (a), χ_0 and χ_∞ (b), ν (c) and χ_0-χ_∞ (d) between 0 and 0.4 T deduced from the generalized Debye fit of the frequency dependence of the real (χ') and imaginary (χ'') components of the ac susceptibility at 2 K, shown in Figure S13, for **4**·3MeOH·0.5H_2O. Solid lines are visual guides.

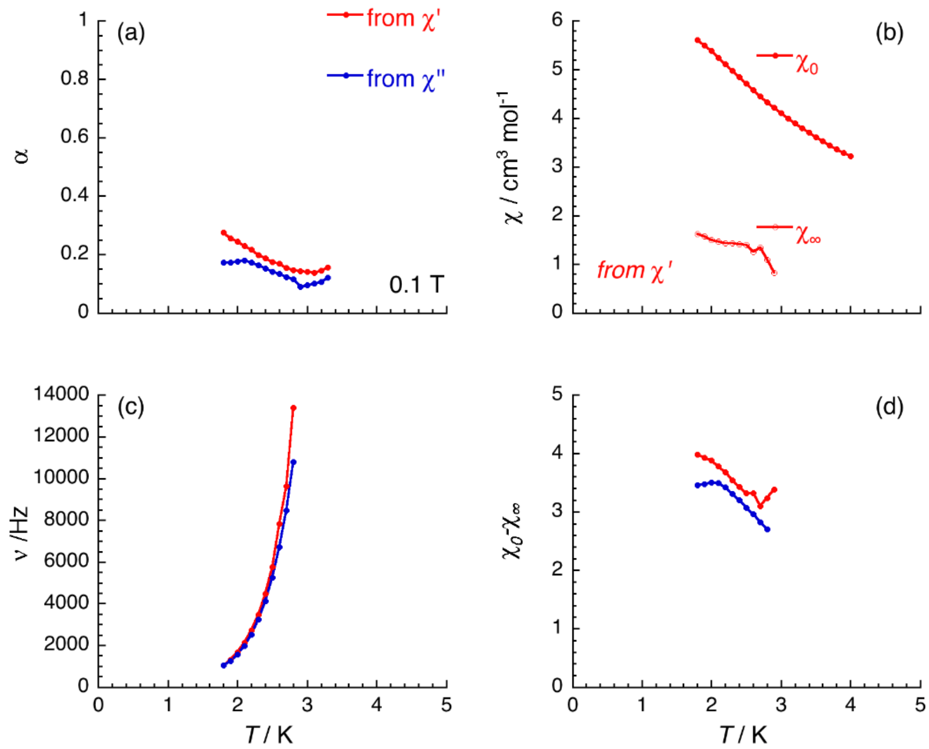

Figure S15. Temperature dependence of the parameters α (a), χ_0 and χ_∞ (b), ν (c) and χ_0-χ_∞ (d) between 1.8 and 3 K deduced from the generalized Debye fit of the frequency dependence of the real (χ') and imaginary (χ'') components of the ac susceptibility at 0.1 T, shown in Figure 9 of the main text, for **4**·3MeOH·0.5H_2O. Solid lines are visual guides.

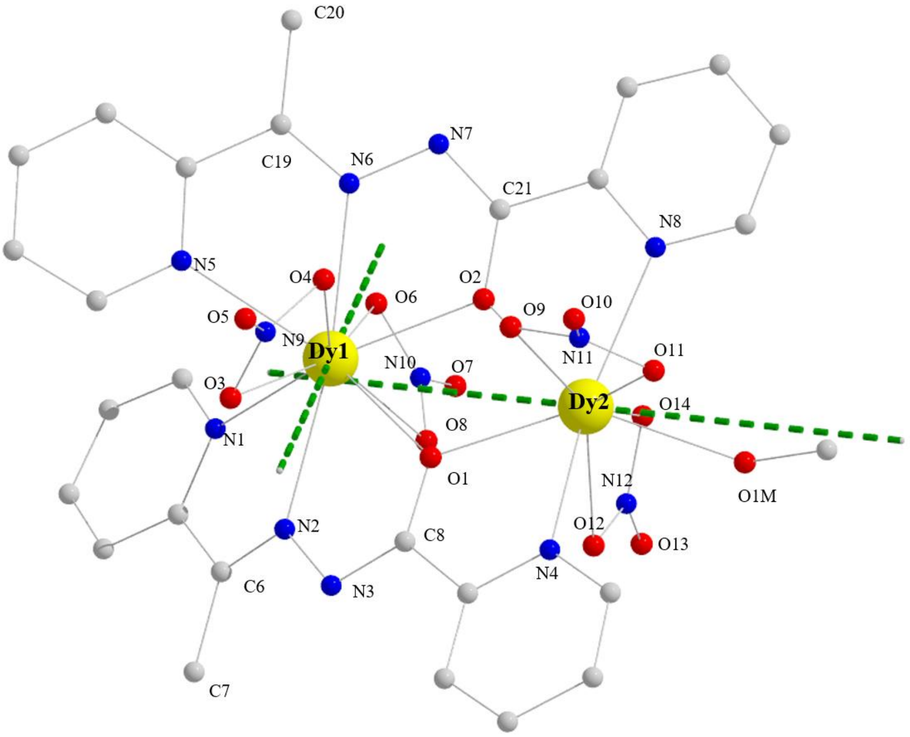

Figure S16. Position of the ground-state magnetic anisotropy axes (dashed green bars) for the two Dy^{III} atoms in the molecule of **3**·2.5MeOH.

Table S1. Continuous Shape Measures (CShM) values for the potential coordination polyhedra of Dy1 in the structure of complex **3**·2.5MeOH.

Ideal Coordination Polyhedron	CShM value[a]
Decagon (DP-10)	34.649
Enneagonal pyramid (EPY-10)	23.939
Octagonal bipyramid (OBPY-10)	16.083
Pentagonal prism (PPR-10)	10.318
Pentagonal antiprism (PARP-10)	10.299
Bicapped cube (BCCU-10)	9.688
Bicapped square antiprism (BCSAPR-10)	4.357
Metabidiminished icosahedron (MBIC-10)	6.864
Augmented tridiminished icosahedron (ATDI-10)	19.747
Sphenocorona (SPC-10)	**2.573**
Staggered dodecahedron (2:6:2)	4.106

[a] The polyhedron with the smallest CShM value (in bold) is the real coordination polyhedron of the Dy1 center for the complex.

Table S2. Continuous Shape Measures (CShM) values for the potential coordination polyhedra of Dy2 in the structure of complex **3**·2.5MeOH.[a.]

Ideal Coordination Polyhedron	CShM value[a]
Enneagon (EP-9)	36.250
Octagonal pyramid (OPY-9)	22.890
Heptagonal bipyramid (HBPY-9)	17.853
Triangular cupola (TC-9)	15.242
Capped cube (JCCU-9)	10.871
Spherical-relaxed capped cube (CCU-9)	9.032
Capped square antiprism (JCSAPR-9)	2.995
Spherical capped square antiprism (CSAPR-9)	**1.759**
Tricapped trigonal prism (JTCTPR-9)	4.508
Spherical tricapped trigonal prism (TCTPR-9)	2.832

[a] The polyhedron with the smallest CShM value (in bold) is the real coordination polyhedron of the Dy2 center for the complex.

© 2020 by the authors. Licensee MDPI, Basel, Switzerland. This article is an open access article distributed under the terms and conditions of the Creative Commons Attribution (CC BY) license (http://creativecommons.org/licenses/by/4.0/).
